# Supplementary material for: Dual vaccination against IL-4 and IL-13 protects against chronic allergic asthma in mice
Source: Nat Commun. 2021 May 11;12:2574. doi: 10.1038/s41467-021-22834-5 (PMC8113315; doi:10.1038/s41467-021-22834-5)
Supplement: Supplementary file 1 — Supplementary Information [file 41467_2021_22834_MOESM1_ESM.pdf]

Dual vaccination against IL-4 and IL-13 protects against chronic allergic asthma in mice

Eva Conde[1,2,3,†], Romain Bertrand[3,†], Bianca Balbino[1,2], Jonathan Bonnefoy[3], Julien Stackowicz[1,2], Noémie Caillot[3], Fabien Colaone[3], Samir Hamdi[3], Raïssa Houmadi[4], Alexia Loste[4], Jasper B.J. Kamphuis[4], François Huetz[1], Laurent Guilleminault[4,5], Nicolas Gaudenzio[4], Aurélie Mougel[4], David Hardy[6], John N. Snouwaert[7], Beverly H. Koller[7], Vincent Serra[3], Pierre Bruhns[1,§], Géraldine Grouard-Vogel[3,§], Laurent L. Reber[1,4,§,*]

[1]Unit of Antibodies in Therapy and Pathology, Institut Pasteur, UMR 1222 INSERM, F-75015 Paris, France. [2]Sorbonne University, ED394, F-75005 Paris, France. [3]Neovacs SA, Paris, France. [4]Toulouse Institute for Infectious and Inflammatory Diseases (Infinity), INSERM UMR1291, CNRS UMR5051, University Toulouse III, 31024 Toulouse, France. [5] Department of respiratory medicine, Toulouse University Hospital, Faculty of Medicine, Toulouse, France. [6]Institut Pasteur, Experimental Neuropathology Unit, Paris, 75015, France. [7]Department of Genetics, University of North Carolina at Chapel Hill, Chapel Hill, NC 27599, USA. [†]These authors contributed equally. [§]These authors jointly supervised this work. [*]Corresponding author.

Email: laurent.reber@inserm.fr

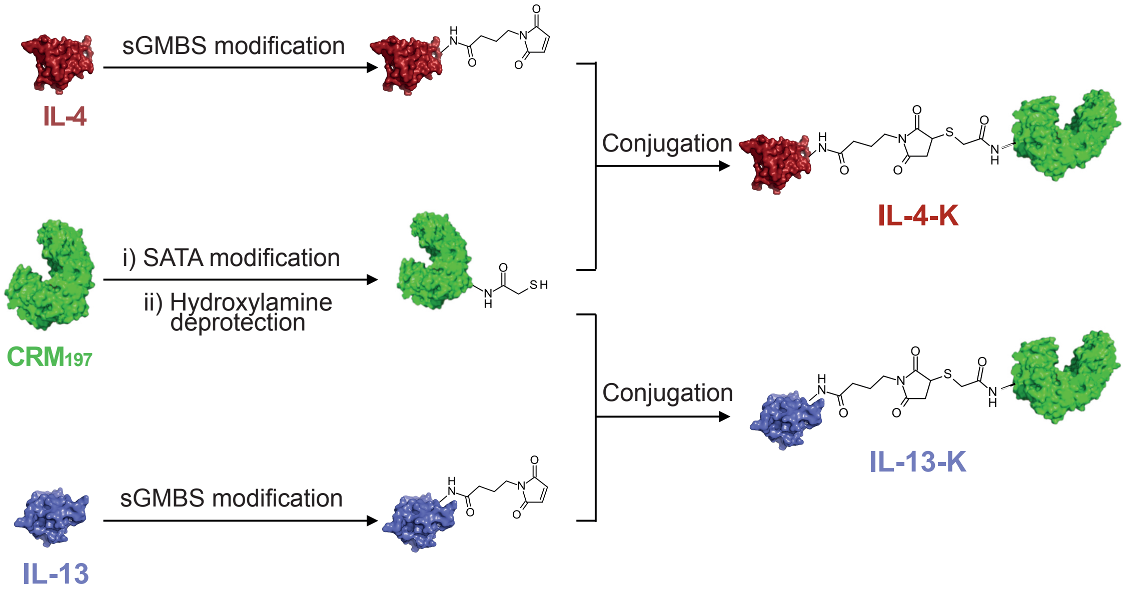

Supplementary Figure 1. Synthesis of IL-4-K and IL-13-K using a thiol-maleimide conjugation. Recombinant mouse IL-4 and IL-13 were modified with N-γ-maleimidobutyryl-oxysuccinimide ester (sGMBS), a maleimide-containing agent reacting with primary amines. Sulfhydryl moieties were introduced on the carrier protein, CRM_{197}, with SATA (N-succinimidyl-S-acetylthioacetate). Functionalized CRM_{197} was added to functionalized IL-4 or IL-13 at a molar ratio of 1:2 (CRM_{197}-IL-4) or 1:4 (CRM_{197}-IL-13), to generate the IL-4 kinoid (IL-4-K) and IL-13-K, respectively.

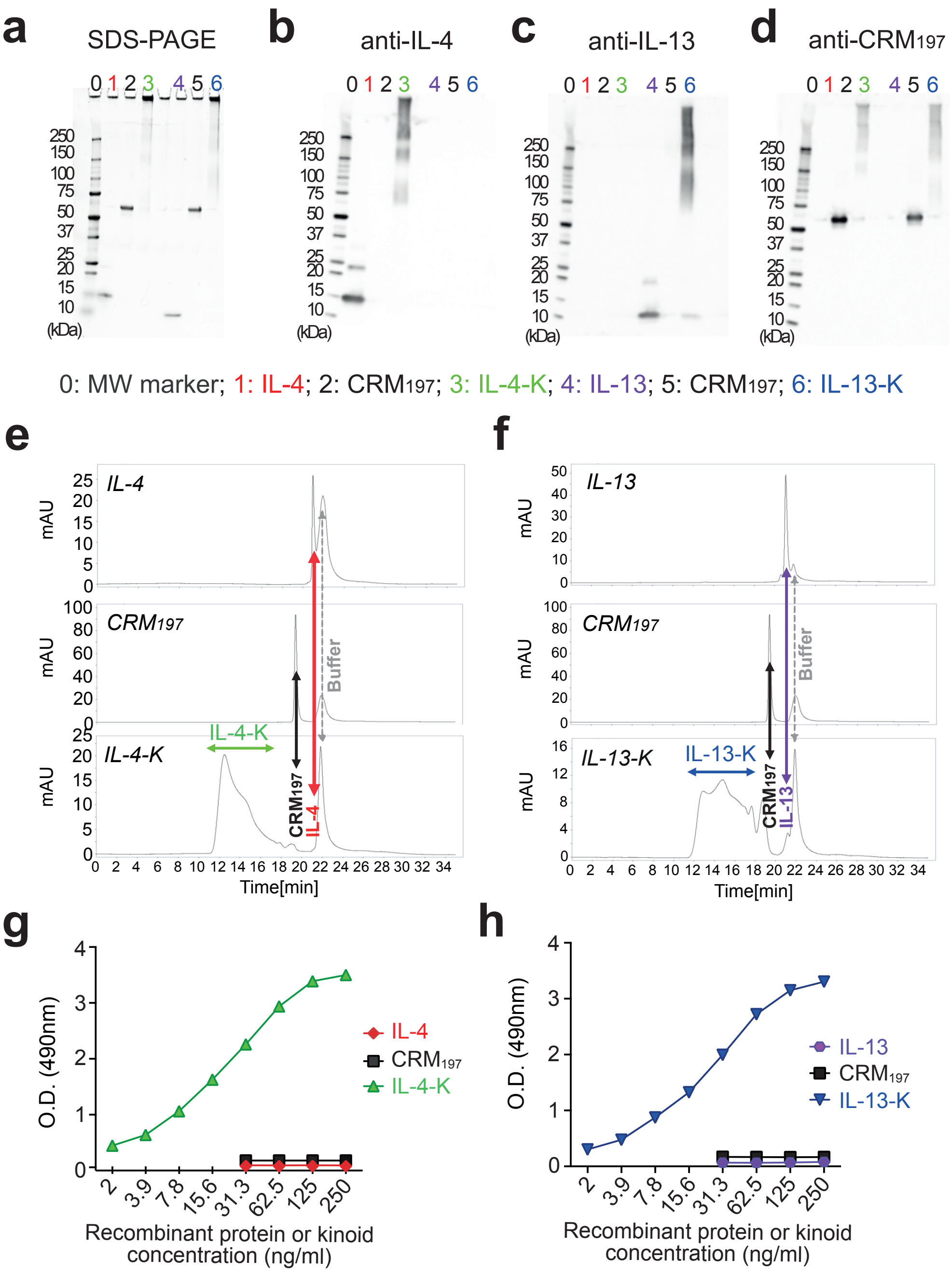

Supplementary Figure 2. Characterization of mouse IL-4-K and IL-13-K. Generation of high molecular weight kinoids upon conjugation of IL-4 or IL-13 to CRM_{197} was confirmed using SDS-PAGE (**a**), western blots against IL-4 (**b**), IL-13 (**c**) or CRM_{197} (**d**), and size exclusion (SE)-HPLC (**e** and **f**). Epitope preservation and antigenicity of IL-4-K and IL-13-K was analyzed by sandwich ELISA using plates coated with anti-CRM_{197} antibodies and revealed with anti-IL-4 (**g**) or anti-IL-13 (**h**) antibodies. Data from a, b, c and d are representative of five independent experiments. Source data are provided in the Source Data file.

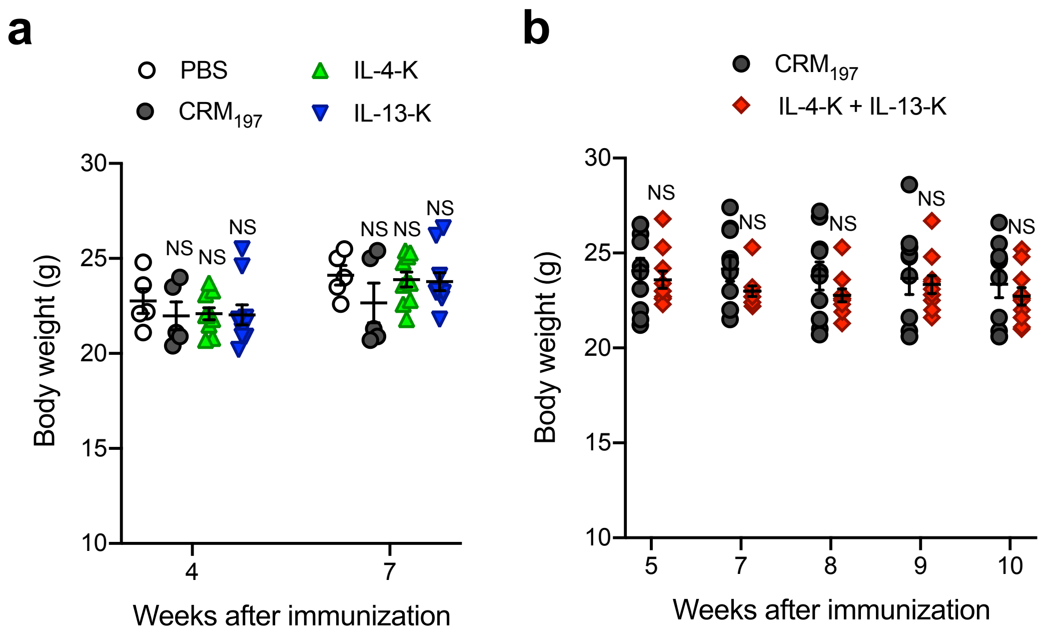

Supplementary Figure 3. Changes in body weight following vaccination with kinoids. Mice were immunized with IL-4-K, IL-13-K, CRM_{197} or PBS as control (**a**), or IL-4-K and IL-13-K in combination or CRM_{197} (**b**), as outlined in Figure 1A. Body weight was measured at the indicated time points after first injection of kinoids. Results show values from individual mice with bars indicating mean values ± SEMs (n=5 mice in PBS and CRM_{197} control groups from (a) or n=10 mice in IL-4-K, IL-13-K, IL-4-K+IL-13-K and CRM_{197} groups), and are pooled from two independent experiments. NS: not significant (P>0.05) *vs*. PBS group in (a) and *vs*. CRM_{197} group in (b). (Mann-Whitney U test). Source data are provided in the Source Data file.

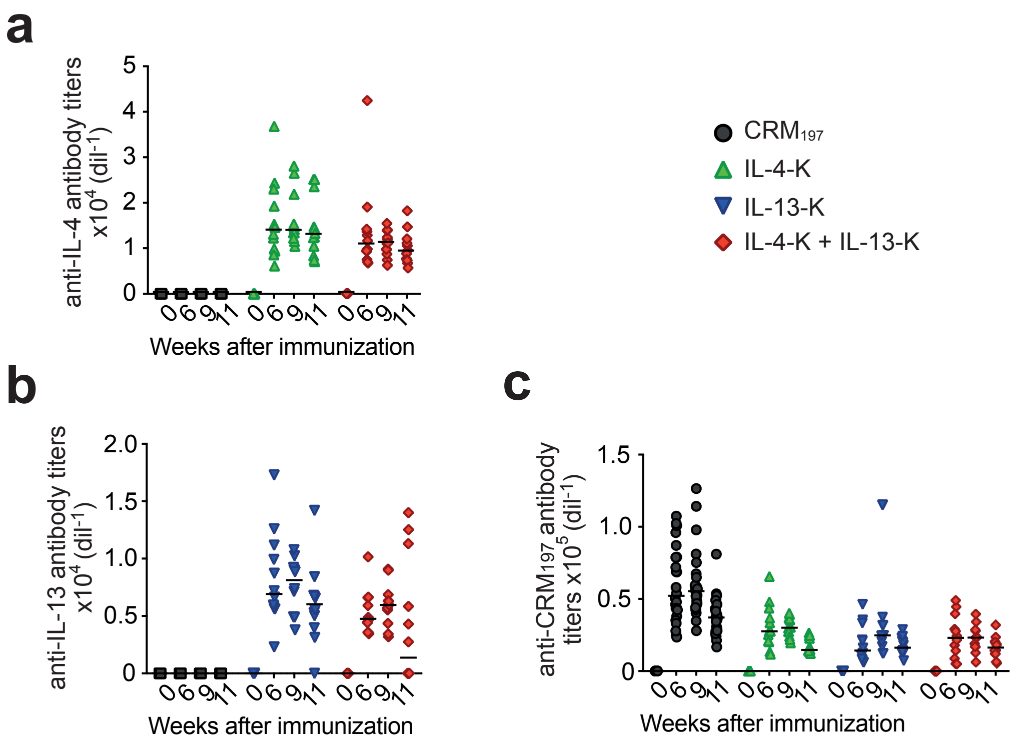

Supplementary Figure 4. Anti-IL-4, anti-IL-13 and anti-CRM197 antibody titers in mice vaccinated with kinoids. a-c. Anti-IL-4 (**a**), anti-IL-13 (**b**) and anti-CRM197 (**c**) antibody titers in sera at 6, 9 and 11 weeks after first injection of IL-4-K and IL-13-K alone or in combination, or CRM197 as a control. Results show values from individual mice with bars indicating medians. Data are from a single experiment with n=12 mice per group, representative of two independent experiments. Source data are provided in the Source Data file.

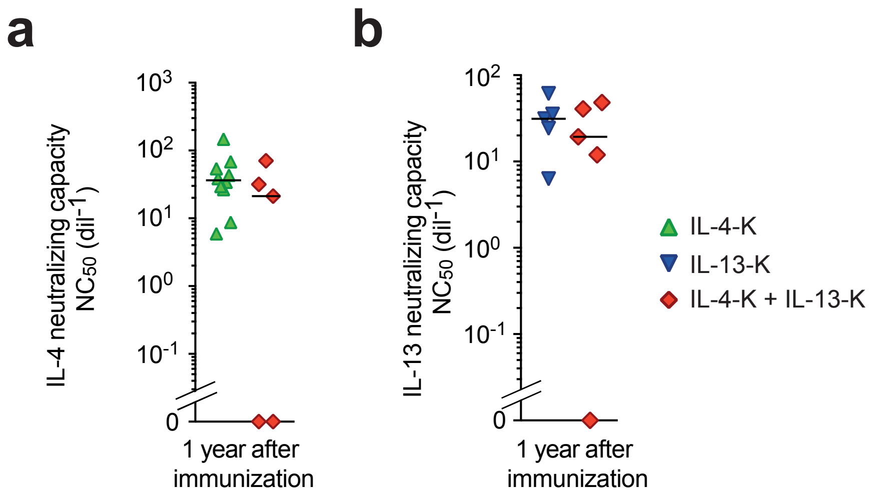

Supplementary Figure 5. Anti-IL-4 and anti-IL-13 neutralizing capacity one year after vaccination. Anti-IL-4 (**a**) and anti-IL-13 (**b**) neutralizing capacity in sera collected one year after the first injection of IL-4-K and/or IL-13-K. Results show values from individual mice with bars indicating medians, and are expressed as the serum dilution factor (dil^{-1}) neutralizing 50 % of IL-4 or IL-13 activity (0 indicates no neutralizing capacity). Data are from a single experiment (*n*=5 m mice in IL-13-K and IL-4-K+IL-13-K groups and *n*=10 mice in IL-4-K group). Source data are provided in the Source Data file.

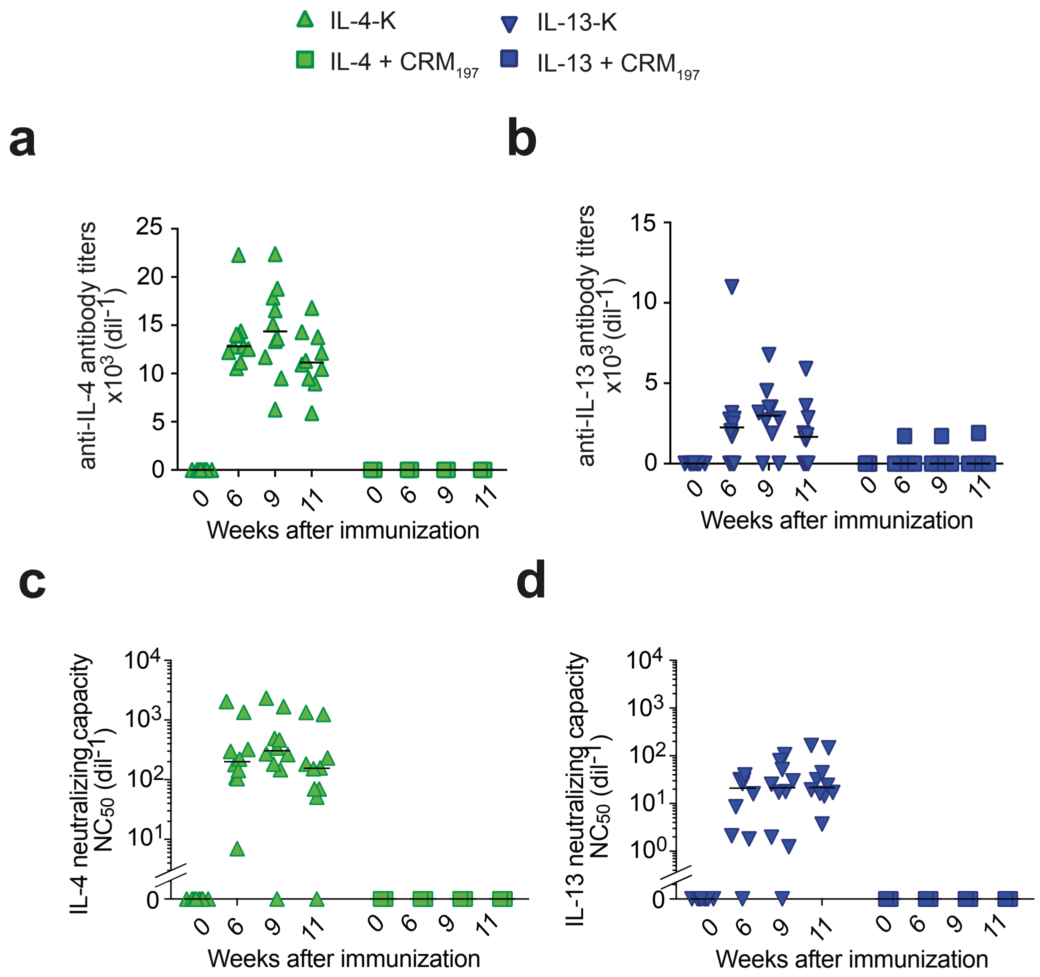

Supplementary Figure 6. Conjugation between IL-4/IL-13 and CRM_{197} is mandatory to mount a potent antibody response. a. Anti-IL-4 antibody titers in sera from mice immunized with IL-4-K or with a mixture of soluble IL-4 and CRM_{197} without prior conjugation. **b**. Anti-IL-13 antibody titers in sera from mice immunized with IL-13-K or with a mixture of soluble IL-13 and CRM_{197} without prior conjugation. Results in **a** and **b** show values from individual mice with bars indicating medians from *n*=10 (IL-4-K or IL-13-K) or *n*=5 (IL-4 + CRM_{197} or IL-13 + CRM_{197}) mice per group. **c**. Anti-IL-4 neutralizing capacity in sera from mice immunized with IL-4-K or with a mixture of soluble IL-4 and CRM_{197} without prior conjugation. **d**. Anti-IL-13 neutralizing capacity in sera from mice immunized with IL-13-K or with a mixture of soluble IL-13 and CRM_{197} without prior conjugation. Results in **c** and **d** show values from individual mice with bars indicating medians from *n*=10 (IL-4-K or IL-13-K) or *n*=5 (IL-4 + CRM_{197} or IL-13 + CRM_{197}) mice per group, and are expressed as the serum dilution factor (dil^{-1}) neutralizing 50 % of IL-4 or IL-13 activity (0 indicates no neutralizing capacity). Source data are provided in the Source Data file.

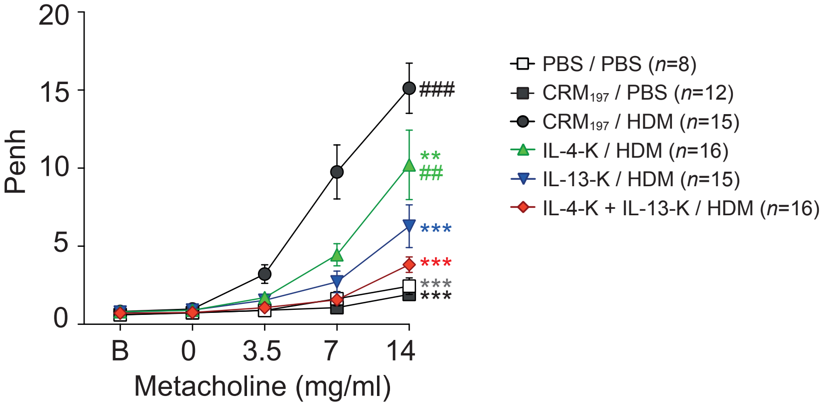

Supplementary Figure 7. Effects of IL-4-K and/or IL-13-K on HDM-induced airway hyperresponsiveness measured using non-invasive plethysmography. Mice were vaccinated with IL-4-K and/or IL-13-K (or PBS or CRM_{197} as controls) followed by sensitization and challenges with HDM (or PBS as a control), as outlined in Figure 1A. Penh responses to increasing doses of methacholine was measured using non-invasive plethysmography 24 hours after the last challenge. Data represent mean values ± SEMs and are pooled from two independent experiments. Adjusted *P*-values calculated using a 2-way ANOVA test followed by a Tukey posttest: ###: $P<0.0001$ and ##: $P=0.0019$ *vs.* CRM_{197}/PBS group; **: $P=0.0027$, ***: $P<0.0001$ *vs.* CRM_{197}/HDM group. Source data are provided in the Source Data file.

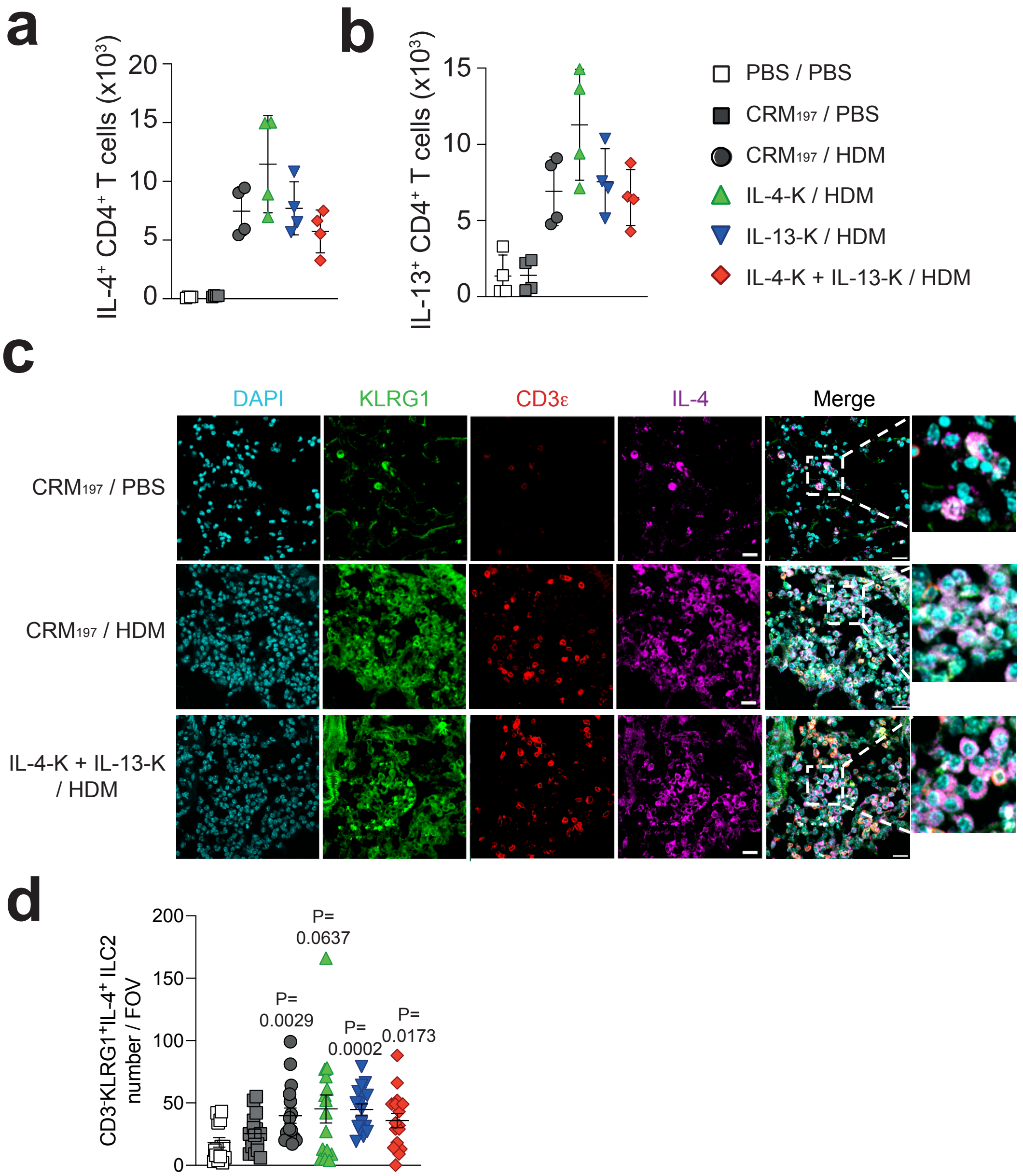

Supplementary Figure 8. Effects of IL-4-K and/or IL-13-K on IL-4$^+$ and IL-13$^+$ CD4 T cells and on IL-4+ ILC2. Mice were vaccinated with IL-4-K and/or IL-13-K (or PBS or CRM$_{197}$ as controls) followed by sensitization and challenges with HDM (or PBS as a control), as outlined in Figure 1A. **a-b.** Number of IL-4$^+$ CD4$^+$ T cells (**a**) IL-13$^+$ CD4$^+$ T cells (**b**) in the lung. Data show values from individual mice with bars indicating mean ± SEM, from a single experiment (*n*=4/group). **c.** Representative lung sections stained with anti-KLRG1 antibodies (green), anti-CD3ε (red), anti-IL-4 (purple) and DAPI (blue), 24 h after the last HDM challenge. Right panels represent magnification of the dashed areas. Scale bars: 30 µm. **d**. Number of ILC2 (identified as KLRG1$^+$CD3ε$^-$ cells) expressing IL-4. Results show values from individual field of view (FOV), with 4 FOV analyzed in each of 4 mice/group. Bars indicate means ± SEMs. *P* values *vs*. PBS/PBS group were calculated using two-tailed Mann-Whitney *U* test. Source data are provided in the Source Data file.

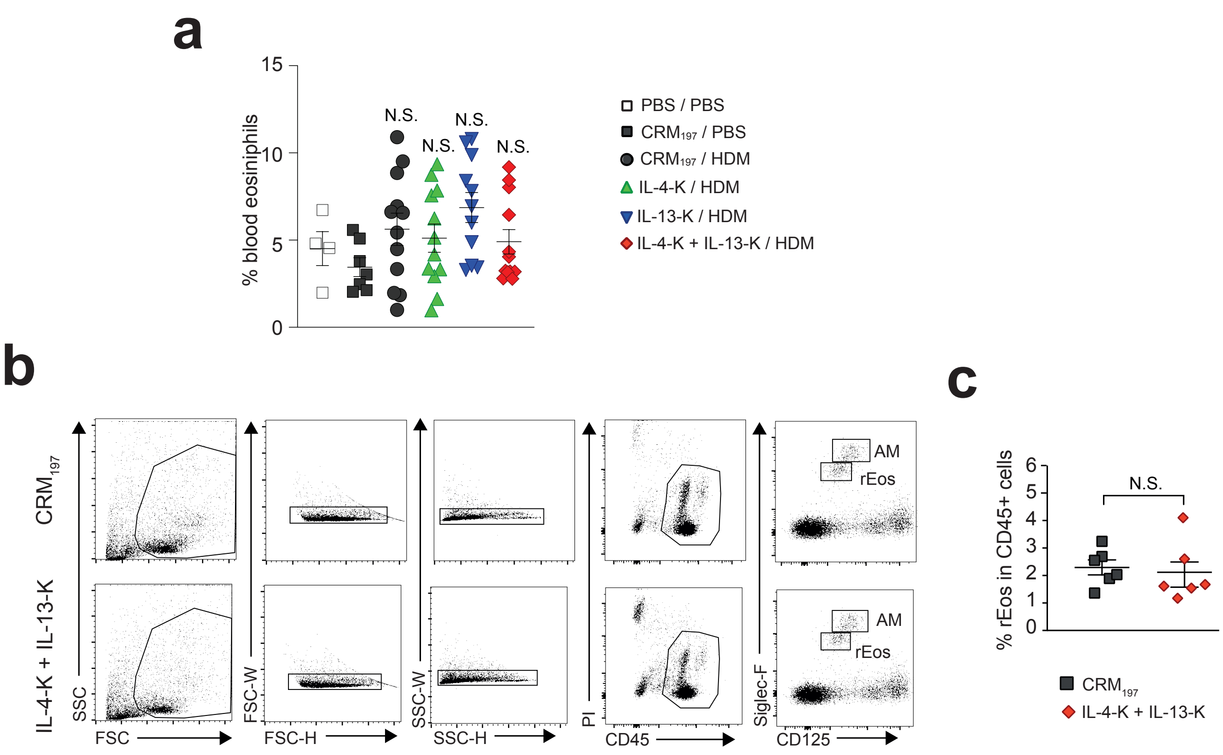

Supplementary Figure 9. Effect of vaccination with IL-4-K and/or IL-13-K on blood eosinophils and lung-resident regulatory eosinophils. a. Mice were vaccinated with IL-4-K and/or IL-13-K (or PBS or CRM_{197} as controls) followed by sensitization and challenge with HDM (or PBS as a control), as outlined in Figure 1A. Percentage of SSC^{high} Siglec-F^{+} blood eosinophils 24 h after the last HDM challenge. Results show values from individual mice with bars indicating means ± SEMs from *n*=4 mice (PBS/PBS control group) or *n*=7 mice (CRM_{197}/PBS control group) or *n*=11 (IL-13-K/HDM group) or *n*=12 mice (CRM_{197}/HDM, IL-4-K/HDM and IL-4-K+IL-13-K/HDM groups) pooled from two independent experiments. **b.** Dot plot of lung leukocytes showing strategy to gate regulatory eosinophils (rEos) according to Siglec-F and CD125 expression (as described previously[23]). AM: alveolar macrophages; FSC, forward scatter; SSC, side scatter; PI: propidium iodide. **c**. Percentage of regulatory eosinophils among resident $CD45^{+}$ lung leukocytes in mice vaccinated with IL-4-K and IL-13-K or CRM_{197} as a control. In a and c, results show values from individual mice with bars indicating means ± SEMs from *n*=6 mice per group pooled from two independent experiments. N.S.: not significant; $P > 0.05$ using two-tailed Mann-Whitney *U* test. Source data are provided in the Source Data file.

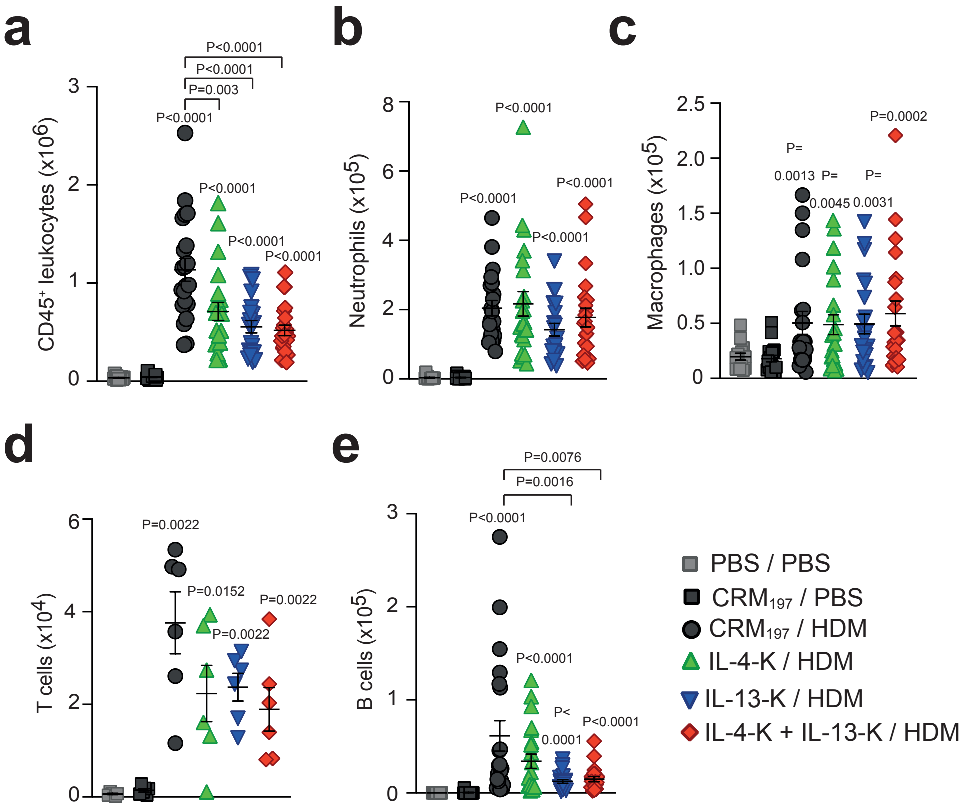

Supplementary Figure 10. Effects of IL-4-K and/or IL-13-K on BAL leukocytes in an asthma model. a-e. Numbers of CD45$^+$ total leukocytes (**a**), CD45$^+$, CD11c$^-$, B220$^-$, CD3ε$^-$, Ly6G$^+$, CD11b$^+$ neutrophils (**b**), CD45$^+$, CD11c$^+$, Siglec-F$^+$, CD11b$^+$ macrophages (**c**), CD45$^+$, CD11c$^-$, CD3ε$^+$ T cells (**d**) and CD45$^+$, CD11c$^-$, B220$^+$ B cells (**e**) in BAL fluid 24 h after the last HDM challenge. Data show values from individual mice with bars indicating mean ± SEM, from a single experiment (in d; *n*=6/group) or pooled from three independent experiments. In a-c and e, *n*=12 mice (PBS/PBS group), *n*=16 mice (CRM/PBS group), *n*=19 mice (IL-13-K/HDM group) or *n*=20 mice (IL-4-K and IL-4-K+IL-13-K groups. *P* values were calculated using two-tailed Mann-Whitney *U* test *vs*. CRM/PBS group or *vs*. indicated group. Source data are provided in the Source Data file.

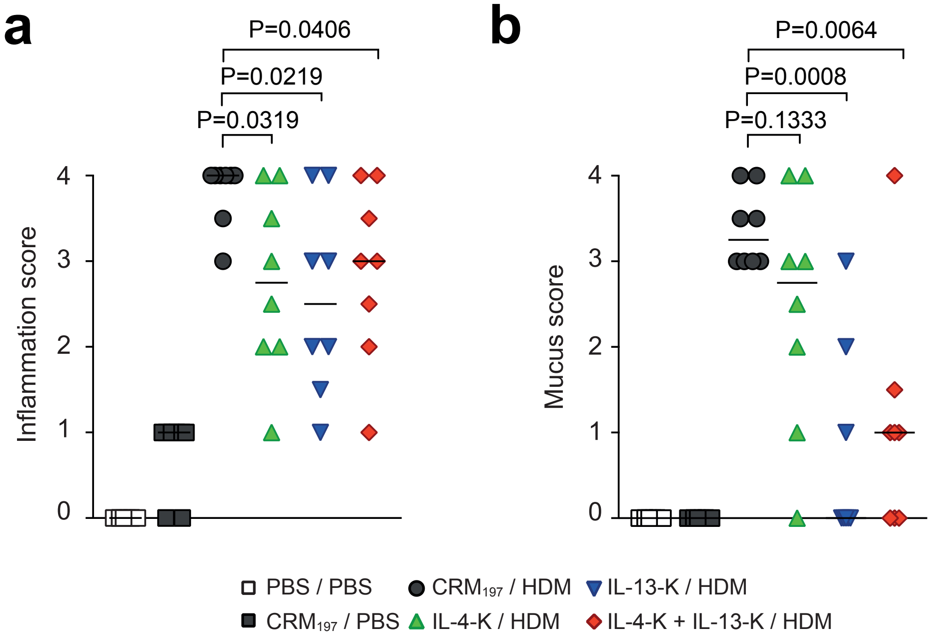

Supplementary Figure 11. Effects of IL-4-K and/or IL-13-K on lung inflammation and mucus production in an asthma model. Mice were vaccinated with IL-4-K and/or IL-13-K (or PBS or CRM_{197} as controls) followed by sensitization and challenge with HDM (or PBS as a control), as outlined in Figure 1a. **a**. The extent of leukocyte infiltration was scored in the H&E-stained lung tissue sections shown in Fig. 1h obtained 24 h after the last challenge with HDM. **b**. The extent of mucus production was scored according to the percentage of goblet cells in bronchial epithelium from the PAS-stained lung tissue sections shown in Fig. 1i obtained 24 h after the last challenge with HDM. Data show values from individual mice with bars indicating median, from a single experiment with n=8 mice per group. P values vs. CRM_{197}/HDM group were calculated using two-tailed Mann-Whitney U test. Source data are provided in the Source Data file.

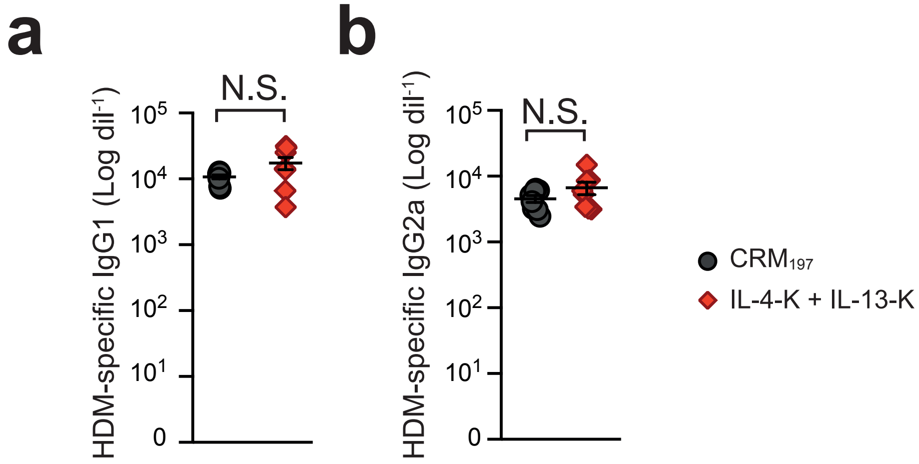

Supplementary Figure 12. Levels of HDM-specific IgG1 and IgG2a in mice vaccinated with IL-4-K and IL-13-K. Levels of HDM-specific IgG1 (**a**) and HDM-specific IgG2a (**b**) 24 h after the last HDM challenge in mice vaccinated with IL-4-K and IL-13-K, or CRM_{197} alone as a control. Results show values from individual mice with bars indicating mean ± SEM. Data are from a single experiment with n=8 mice per group, representative of two independent experiments. N.S.: not significant ($P > 0.05$ using two-tailed Mann-Whitney U test). Source data are provided in the Source Data file.

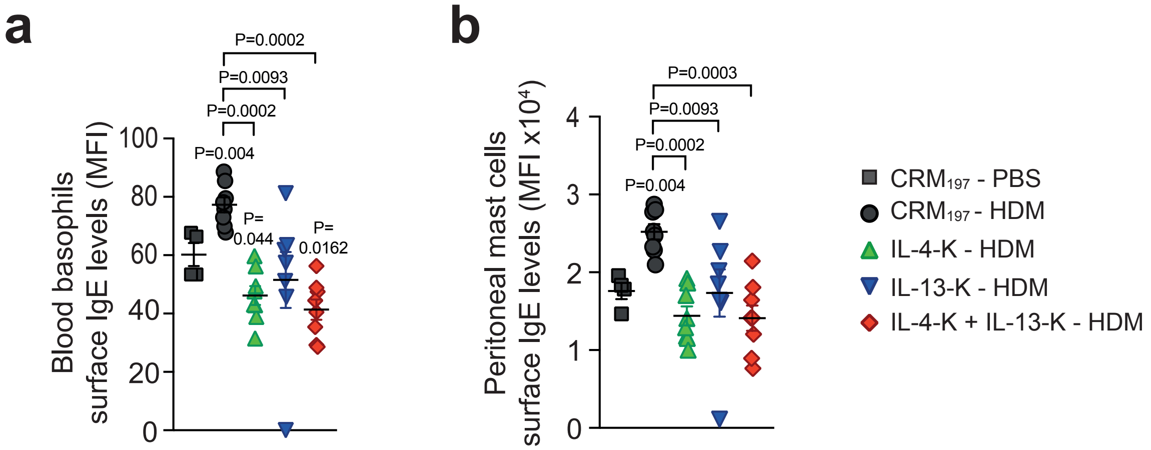

Supplementary Figure 13. Effects of IL-4-K and/or IL-13-K on surface IgE levels in blood basophils and peritoneal mast cells. Mice were vaccinated with IL-4-K and/or IL-13-K (or PBS or CRM_{197} as controls) followed by sensitization and challenge with HDM (or PBS as a control), as outlined in Figure 1**a**. Levels of IgE on the surface of IgE^+ $CD49b^+$ blood basophils (MFI: mean fluorescence intensity) (a), and levels of IgE on the surface of $CD117^+$ peritoneal mast cells (MFI) (b) 24 h after the last HDM challenge. Results show values from individual mice with bars indicating means ± SEMs from *n*=4 mice (CRM_{197}/PBS control group), *n*=7 mice (IL-13-K/HDM) or *n*=8 mice (IL-4-K/HDM, IL-4-K+IL-13-K/HDM) pooled from two independent experiments. *P* values vs. CRM_{197}/PBS group or *vs*. indicated group were calculate using two-tailed Mann-Whitney U test. Source data are provided in the Source Data file.

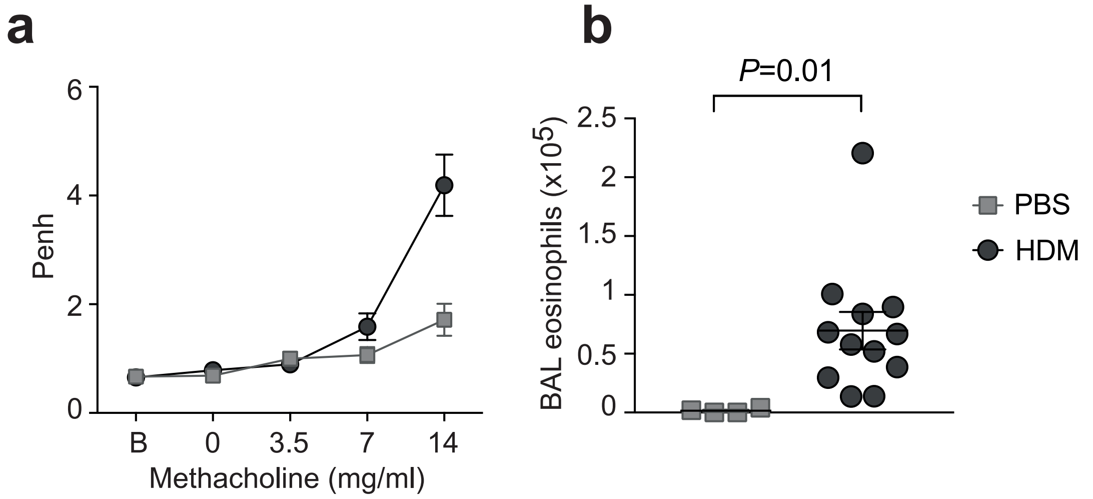

Supplementary Figure 14. Evidence of established airway hyperreactivity and eosinophilia before the start of therapeutic vaccination. Mice were sensitized and challenged with HDM or PBS as a control, as outlined in Figure 3A. Penh responses to increasing doses of methacholine measured using non-invasive plethysmography (**a**), and numbers of eosinophils in BAL fluid (**b**) 24 hours after the third challenge (*i.e.* immediately before vaccination with IL-4-K and IL-13-K). Data represent mean values ± SEMs from *n*=4 mice (PBS group) or *n*=12 mice (HDM group) pooled from two independent experiments. *P* values were calculated using two-tailed Mann-Whitney *U* test. Source data are provided in the Source Data file.

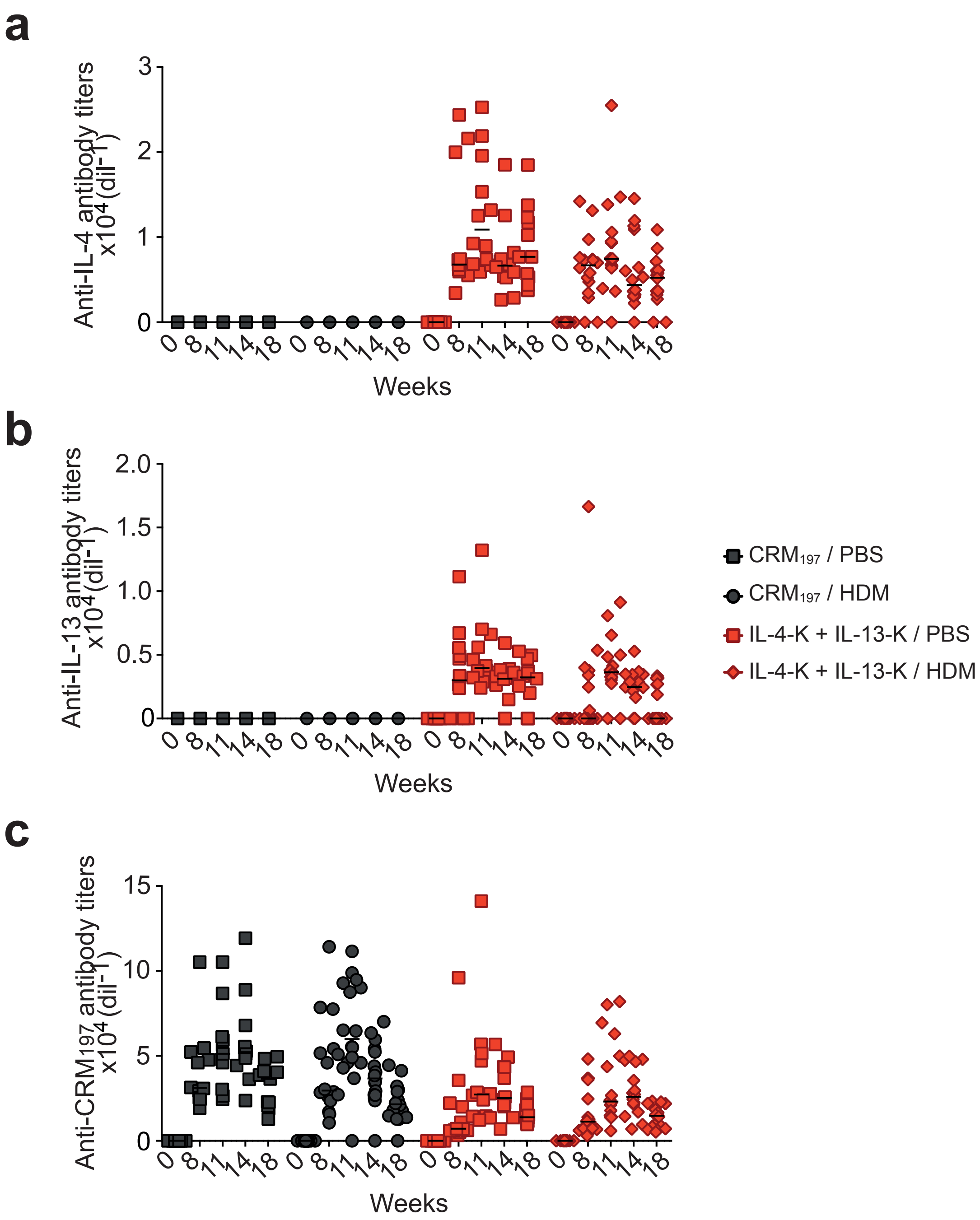

Supplementary Figure 15. Anti-IL-4, anti-IL-13 and anti-CRM$_{197}$ antibody titers upon therapeutic dual vaccination with IL-4-K and IL-13-K. Mice were sensitized and challenged with HDM extract (or PBS as a control). After the third challenge, mice were vaccinated with IL-4-K and IL-13-K in combination (or CRM$_{197}$ as control), as outlined in Figure 3A. Anti-IL-4 (**a**), anti-IL-13 (**b**) and anti-CRM$_{197}$ antibody titers (**c**) in sera from mice at indicated time points. Results show values from individual mice pooled from two independent experiments with bars indicating median (n=12 for all PBS groups and n=16 for all HDM groups). Source data are provided in the Source Data file.

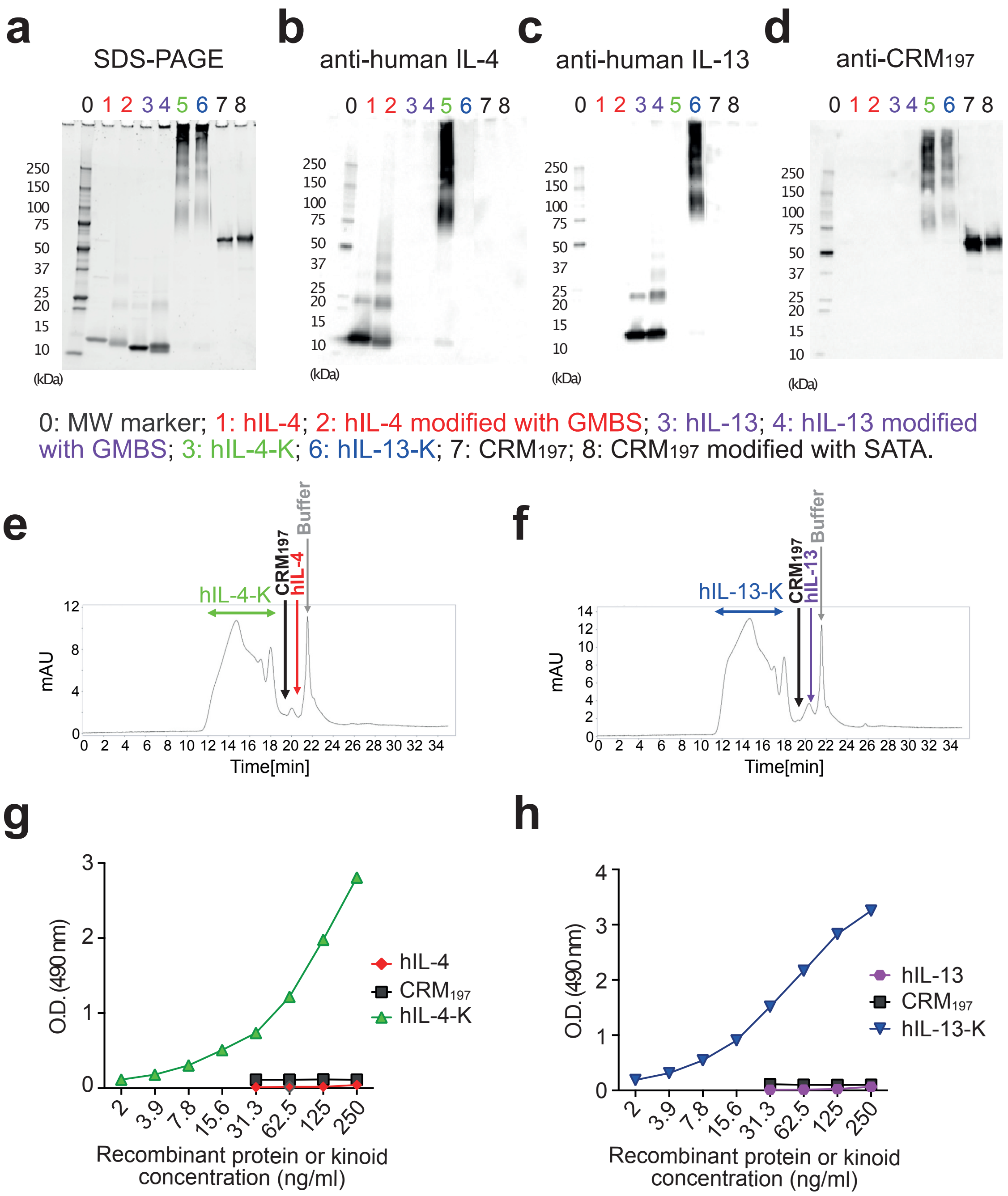

Supplementary Figure 16. Characterization of human IL-4-K and IL-13-K. Generation of high molecular weight kinoids upon conjugation of human IL-4 or human IL-13 to CRM_{197} was confirmed using SDS-PAGE (**a**), western blots against human IL-4 (**b**), human IL-13 (**c**) or CRM_{197} (**d**), and size exclusion (SE)-HPLC (**e** and **f**). Epitope preservation and antigenicity of human IL-4-K (hIL-4-K) and hIL-13-K was analyzed by sandwich ELISA using plates coated with anti-CRM_{197} antibodies and revealed with anti-human IL-4 (**g**) or anti-human IL-13 (**h**) antibodies. Data from a-d are representative of 3 independent experiments. Source data are provided in the Source Data file.

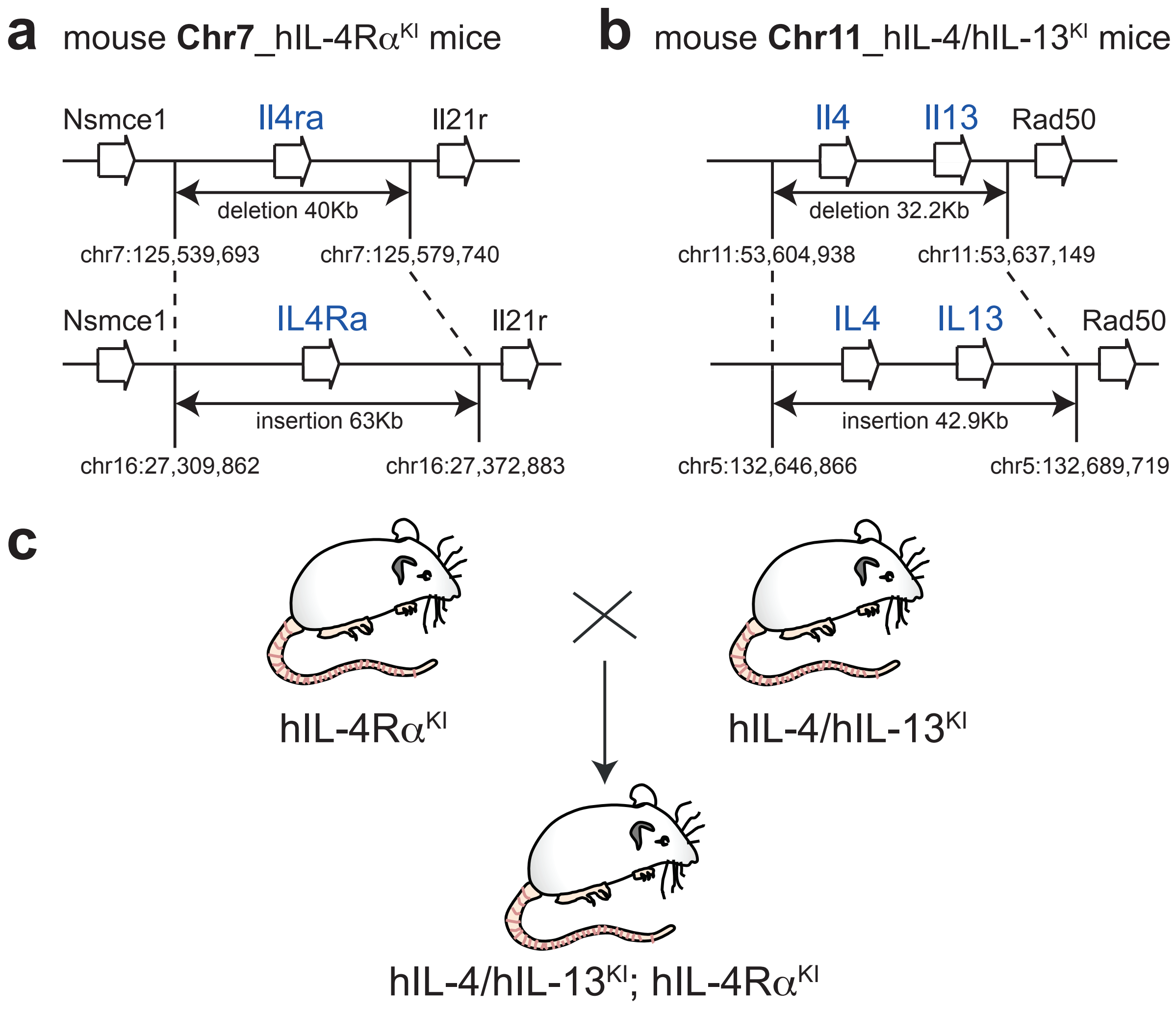

Supplementary Figure 17. Generation of hIL-4/hIL-13^{KI}; hIL-4Rα^{KI} mice. a-b. Humanization of the mouse Il4ra locus (**a**) and il4/il13 locus (**b**). Representations are not drawn to scale. **c**. Breeding scheme to obtain hIL-4/hIL-13^{KI}; hIL-4Rα^{KI} mice.

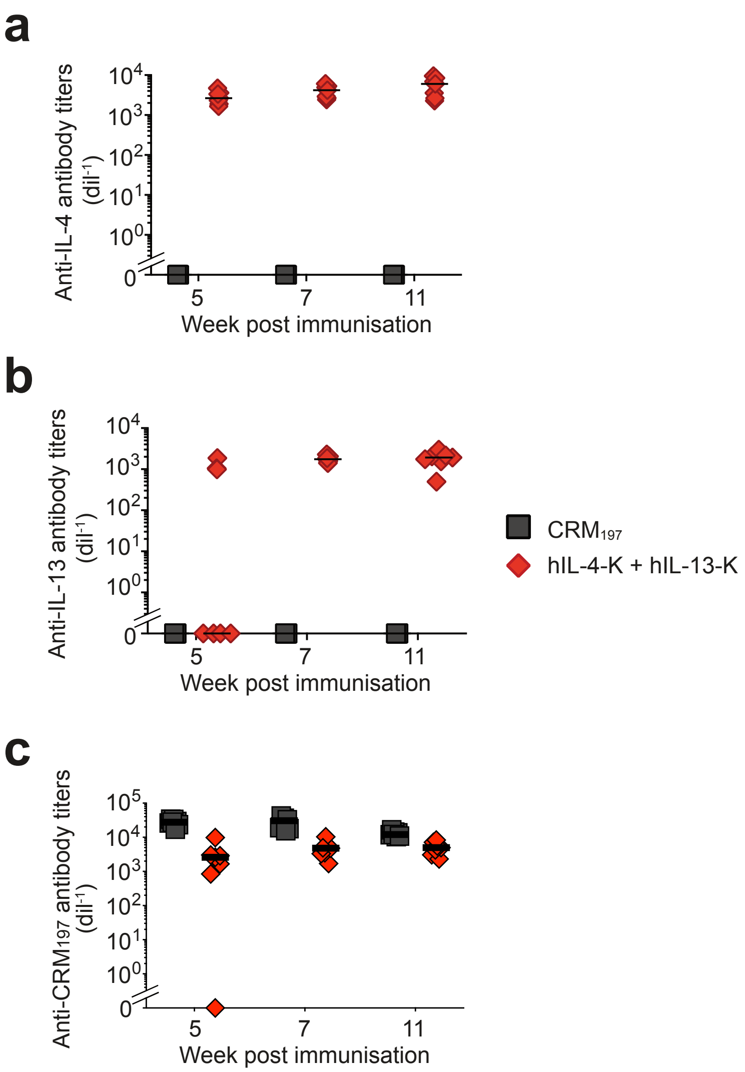

Supplementary Figure 18. Anti-hIL-4, anti-hIL-13 and anti-CRM197 antibody titers upon dual vaccination with human IL-4 and IL-13 kinoids in IL-4/IL-13^{KI}; IL-4Rα^{KI} humanized mice. IL-4/IL-13^{KI}; IL-4Rα^{KI} humanized mice were vaccinated with hIL-4-K and hIL-13-K in combination (or CRM197 as control), as described in Fig. 4a. Anti-human IL-4 (**a**), anti-human IL-13 (**b**) and anti-CRM197 (**c**) antibody titers in sera at the indicated time-points. Results show values from individual mice with bars indicating medians (*n*=7 mice per group). Source data are provided in the Source Data file.

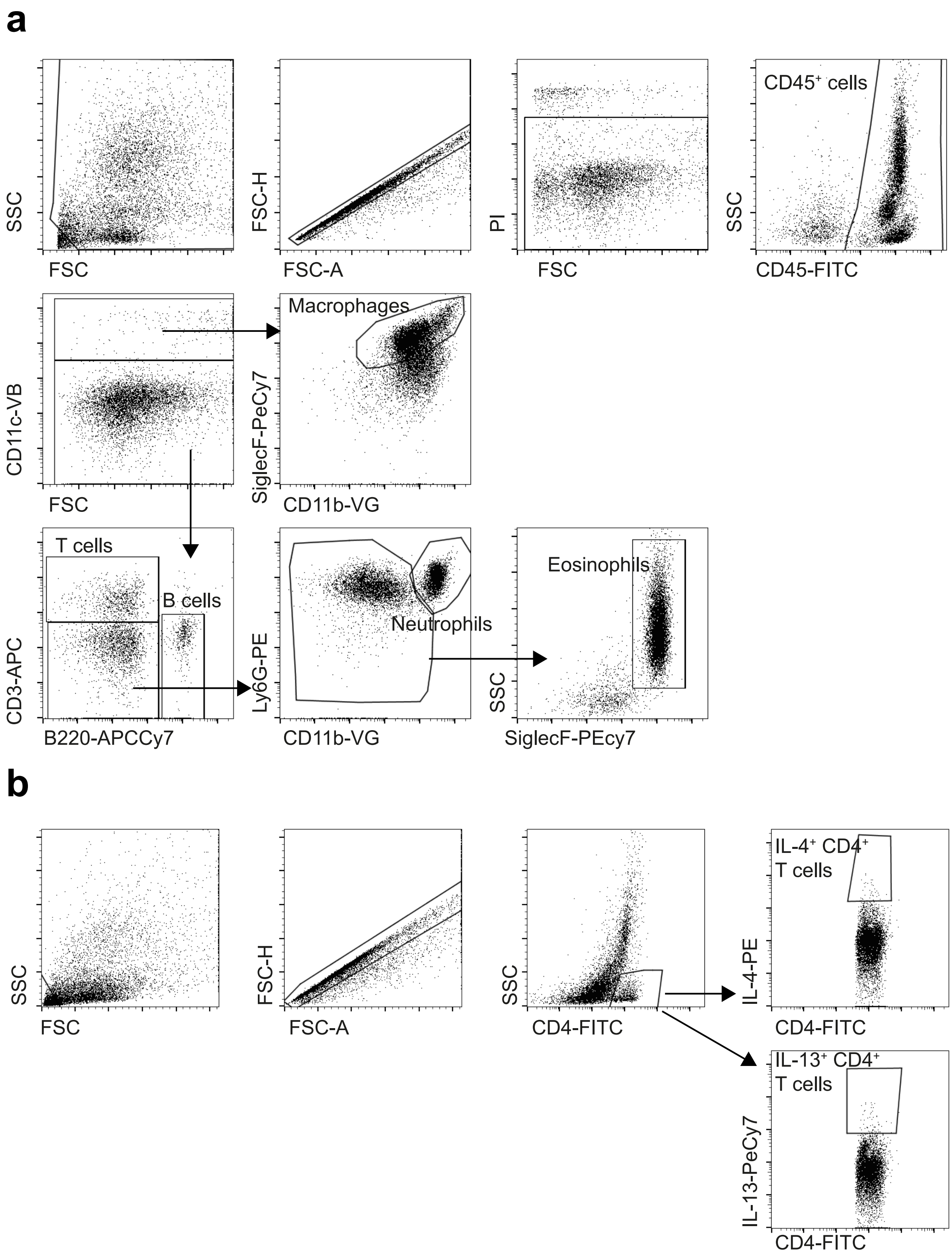

Supplementary Figure 19. Gating strategy for the analysis of leukocyte populations in BAL and lung cell suspensions (**a**) and intracellular IL-4 and IL-13 levels in lung CD4+ T cells (**b**) by flow cytometry.

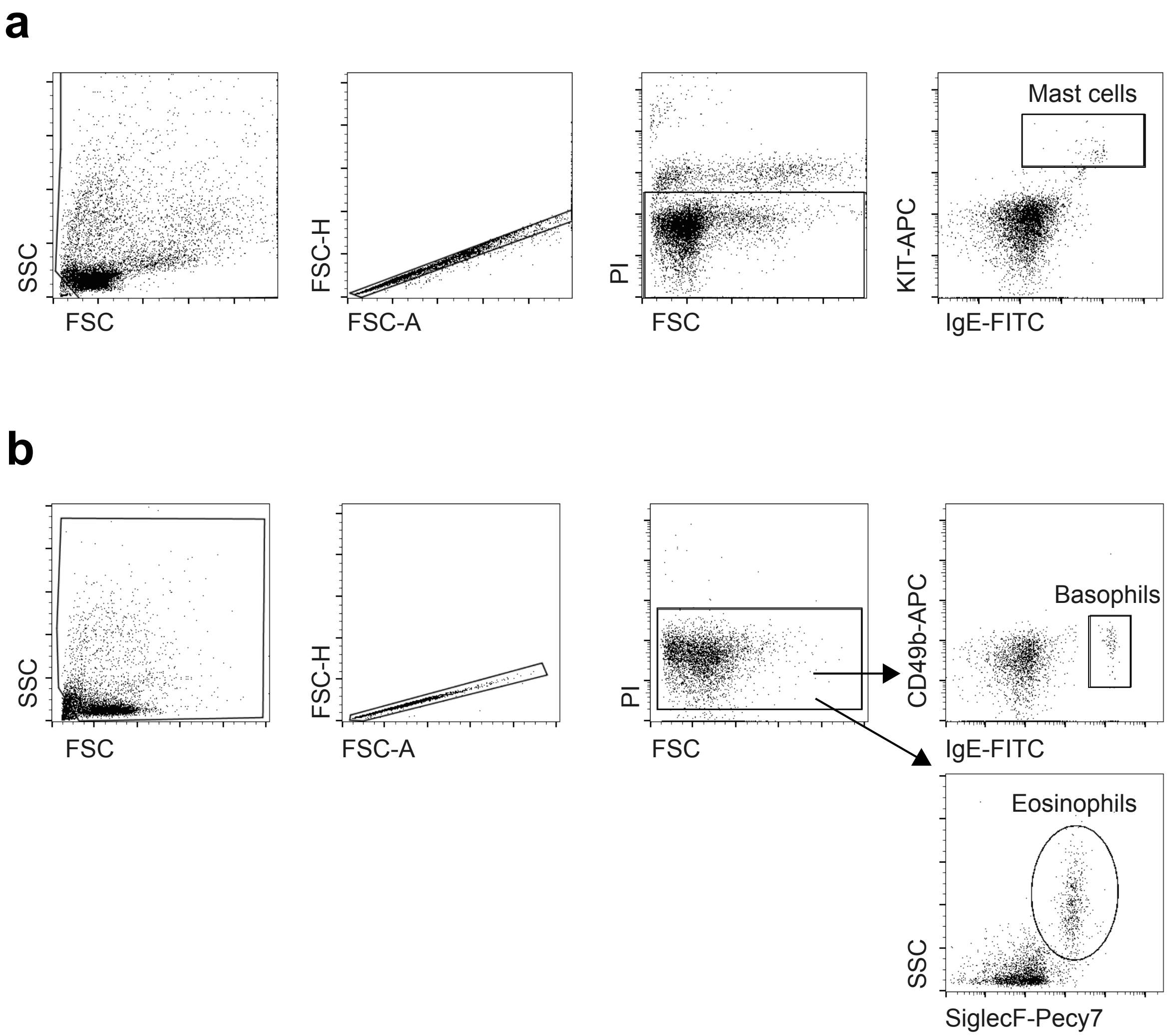

Supplementary Figure 20. Gating strategy for the analysis of peritoneal mast cells (**a**) and blood basophils and eosinophils (**b**) by flow cytometry.

ANTIBODY	SOURCE	CLONE	IDENTIFIER	Dilution
anti-mouse IL-4 polyclonal goat IgG	R&D systems	n/a	AF-404-NA	0.1 µg/ml
anti-human IL-4 polyclonal goat IgG	R&D systems	n/a	AF-204-NA	0.1 µg/ml
anti-mouse IL-13 polyclonal goat IgG	R&D systems	n/a	AF-413-NA	0.1 µg/ml
anti-human IL-13 polyclonal goat IgG	R&D systems	n/a	AF-213-NA	0.1 µg/ml
anti-diphtheria toxin polyclonal goat IgG	AbD serotec	n/a	3710-0956	860 ng/ml
monoclonal mouse anti-diphtheria toxin IgG1	AbD serotec	8G1	3710-0100	1 µg/ml
anti-human IL-4 monoclonal mouse IgG1	R&D systems	3007	MAB304	1 µg/ml
anti-human IL-13 monoclonal mouse IgG1	R&D systems	32116	MAB213	1 µg/ml
biotinylated anti-mouse IL-4 polyclonal goat IgG	R&D systems	n/a	BAF-404	250 ng/ml
biotinylated anti-human IL-4 polyclonal goat IgG	R&D systems	n/a	BAF-204	250 ng/ml
biotinylated anti-mouse IL-13 polyclonal goat IgG	R&D systems	n/a	BAF-413	250 ng/ml
biotinylated anti-human IL-13 polyclonal goat IgG	R&D systems	n/a	BAF-213	250 ng/ml
anti-Ly6G-PE rat IgG_{2a}	BD Pharmingen	1A8	561104	1:100
anti-CD3-APC armenian hamster IgG_1	BD Pharmingen	145-2C11	561826	1:100
anti-CD4-FITC Rat IgG2bk	Miltenyi Biotec	GK1.5	130-120-819	1:50
anti-IL-4-PE rat IgG2b	Miltenyi Biotec	BVD4-1D11	130-103-016	1:20
anti-mouse IL-4-APC Rat IgG1k	eBioscience	11B11	53-7041-82	10 µg/ml
anti-IL-13-PE-Cyanine7 Rat IgG1	Fisher Scientific	eBio13A	15538636	1:200
anti-KLRG1-AF488 Hamster $IgG_{2\kappa}$	BD Pharmingen	2F1	562190	10 µg/ml
anti-CD3-AF532	eBioscience	17A2	58-0032-82	2 µg/ml
anti-CD45-FITC human IgG_1	Miltenyi Biotec	REA737	130-110-658	1:100
anti-CD45-VB human IgG1	Miltenyi Biotec	REA737	130-110-802	1:200
anti-Siglec-F-PECy7 human IgG_1	Miltenyi Biotec	REA798	130-112-334	1:100
anti-CD11b-VG recombinant human IgG_1	Miltenyi Biotec	REA713	130-110-559	1:100
anti-B220-APC rat IgG_{2a}	Miltenyi Biotec	RA3-6B2	130-102-259	1:100
anti-CD11c-VB, hamster IgG	Miltenyi Biotec	N418	130-102-797	1:100
anti-CD49b-APC, Rat IgM	eBioscience	DX5	17-5971-82	1:100
anti-IgE-FITC, Rat IgG_1	BD Pharmingen	R35-72	553415	1:50

anti-CD125-PE, Rat IgG1	BD Pharmingen	T21	558488	1:100
Purified NA/LE Rat Anti-Mouse CD124	BD Pharmingen	mIL4R-M1	552288	10 µg/mL
anti-Siglec-F-Alexa647, Rat IgG2a	BD Pharmingen	E50-2440	562680	1:100
anti-c-KIT APC	eBioscience	2B8	17-1171-82	1:200
rabbit polyclonal HRP-conjugated anti-mouse IgG	Invitrogen	n/a	61-6520	1:5000
Goat polyclonal Anti-Mouse IgG1 Human ads-HRP	Southern Biotech	n/a	1070-05	1:4000
Goat anti-Rat IgG (H+L), Alexa Fluor 594	Invitrogen	n/a	A11007	1:150
Goat polyclonal Anti-Mouse IgG2a Human ads-HRP	Southern Biotech	n/a	1080-05	1:4000
goat polyclonal anti-mouse IgE antibody	Bio-rad	n/a	STAR110	2 µg/mL
goat polyclonal HRP-conjugated anti-mouse IgG	Bethyl Laboratories	n/a	A90-131P	1:5000
anti-IgE antibody coupled to AF488, Rat IgG1, κ	Biolegend	RME-1	406910	10 µg/ml

Supplementary Table 1. List of antibodies used in this study.
