# Supplementary material for: Dual vaccination against IL-4 and IL-13 protects against chronic allergic asthma in mice
Source: Nat Commun. 2021 May 11;12:2574. doi: 10.1038/s41467-021-22834-5 (PMC8113315; doi:10.1038/s41467-021-22834-5)
Supplement: Supplementary file 3 — Reporting Summary [file 41467_2021_22834_MOESM3_ESM.pdf]

nature research

Corresponding author(s): Laurent L. Reber
Last updated by author(s): Mar 12, 2021

Reporting Summary

Nature Research wishes to improve the reproducibility of the work that we publish. This form provides structure for consistency and transparency in reporting. For further information on Nature Research policies, see our Editorial Policies and the Editorial Policy Checklist.

Statistics

For all statistical analyses, confirm that the following items are present in the figure legend, table legend, main text, or Methods section.

n/a	Confirmed	
☐	☒	The exact sample size (*n*) for each experimental group/condition, given as a discrete number and unit of measurement
☐	☒	A statement on whether measurements were taken from distinct samples or whether the same sample was measured repeatedly
☐	☒	The statistical test(s) used AND whether they are one- or two-sided<br>*Only common tests should be described solely by name; describe more complex techniques in the Methods section.*
☒	☐	A description of all covariates tested
☒	☐	A description of any assumptions or corrections, such as tests of normality and adjustment for multiple comparisons
☐	☒	A full description of the statistical parameters including central tendency (e.g. means) or other basic estimates (e.g. regression coefficient) AND variation (e.g. standard deviation) or associated estimates of uncertainty (e.g. confidence intervals)
☐	☒	For null hypothesis testing, the test statistic (e.g. *F*, *t*, *r*) with confidence intervals, effect sizes, degrees of freedom and *P* value noted<br>*Give P values as exact values whenever suitable.*
☒	☐	For Bayesian analysis, information on the choice of priors and Markov chain Monte Carlo settings
☒	☐	For hierarchical and complex designs, identification of the appropriate level for tests and full reporting of outcomes
☒	☐	Estimates of effect sizes (e.g. Cohen's *d*, Pearson's *r*), indicating how they were calculated

Our web collection on statistics for biologists contains articles on many of the points above.

Software and code

Policy information about availability of computer code

Data collection	IOX base 8c/RF-8a (0796) software, FlexiVent software FlexiWare 8.0
Data analysis	FlowJo 10.4.2, GraphPad Prism 7.0, Microsoft Excel 16.16.23, Zen Software, ImageJ (1.53c), Datanalyst (DATA 4238)

For manuscripts utilizing custom algorithms or software that are central to the research but not yet described in published literature, software must be made available to editors and reviewers. We strongly encourage code deposition in a community repository (e.g. GitHub). See the Nature Research guidelines for submitting code & software for further information.

Data

Policy information about availability of data

All manuscripts must include a data availability statement. This statement should provide the following information, where applicable:

- Accession codes, unique identifiers, or web links for publicly available datasets
- A list of figures that have associated raw data
- A description of any restrictions on data availability

All data generated or analyzed during this study are included in this published article (and its Supplementary information files). Source data are provided with this paper.

Field-specific reporting

Life sciences study design

All studies must disclose on these points even when the disclosure is negative.

Sample size	Sample size for each experiment is indicated in the legend. No statistical tests were used to pre-determine sample size. Sample size was chosen based on previous experiments published by some of the co-authors (Sibilano et al Nat Commun 2016) and comparable studies in literature.
Data exclusions	No data were excluded in this study
Replication	All attemps at replication were successful. Each animal experiment presented in the paper was repeated in multiple animals and the number of replications was stated in the figure legends.<br>Experiments from figure 1 and 2 were repeated three times.<br>Experiments from figure 3 were repeated two times.<br>Experiments from figure 4 were repeated two times<br>Experiments from figure 5 were done once
Randomization	Mice were randomly atributed to a treatment or control group.<br>Allocation was done randomly.<br>All the experiments were done with samples coming from the mice randomly atributed to a treatment or control group.
Blinding	No blinding was possible because experiments required repeated injections of different vaccines. For histology studies, quantification of staining and cell population were determined by an individual unaware of the sample's identity.

Reporting for specific materials, systems and methods

We require information from authors about some types of materials, experimental systems and methods used in many studies. Here, indicate whether each material, system or method listed is relevant to your study. If you are not sure if a list item applies to your research, read the appropriate section before selecting a response.

Materials & experimental systems

n/a	Involved in the study	
☐	☒	Antibodies
☐	☒	Eukaryotic cell lines
☒	☐	Palaeontology and archaeology
☐	☒	Animals and other organisms
☒	☐	Human research participants
☒	☐	Clinical data
☒	☐	Dual use research of concern

Methods

n/a	Involved in the study	
☒	☐	ChIP-seq
☐	☒	Flow cytometry
☒	☐	MRI-based neuroimaging

Antibodies

Antibodies used

ANTIBODY SOURCE CLONE IDENTIFIER Dilution
anti-mouse IL-4 polyclonal goat IgG R&D systems n/a AF-404-NA 0.1 µg/ml
anti-human IL-4 polyclonal goat IgG R&D systems n/a AF-204-NA 0.1 µg/ml
anti-mouse IL-13 polyclonal goat IgG R&D systems n/a AF-413-NA 0.1 µg/ml
anti-human IL-13 polyclonal goat IgG R&D systems n/a AF-213-NA 0.1 µg/ml
anti-diphtheria toxin polyclonal goat IgG AbD serotec n/a 3710-0956 860 ng/ml
monoclonal mouse anti-diphtheria toxin IgG1 AbD serotec 8G1 3710-0100 1 µg/ml
anti-human IL-4 monoclonal mouse IgG1 R&D systems 3007 MAB304 1 µg/ml
anti-human IL-13 monoclonal mouse IgG1 R&D systems 32116 MAB213 1 µg/ml
biotinylated anti-mouse IL-4 polyclonal goat IgG R&D systems n/a BAF-404 250 ng/ml
biotinylated anti-human IL-4 polyclonal goat IgG R&D systems n/a BAF-204 250 ng/ml
biotinylated anti-mouse IL-13 polyclonal goat IgG R&D systems n/a BAF-413 250 ng/ml
biotinylated anti-human IL-13 polyclonal goat IgG R&D systems n/a BAF-213 250 ng/ml
anti-Ly6G-PE rat IgG2a BD Pharmingen 1A8 561104 1:100
anti-CD3-APC armenian hamster IgG1 BD Pharmingen 145-2C11 561826 1:100
anti-CD4-FITC Rat IgG2bk Miltenyi Biotec GK1.5 130-120-819 1:50
anti-IL-4-PE rat IgG2b Miltenyi Biotec BVD4-1D11 130-103-016 1:20
anti-mouse IL-4-APC Rat IgG1k eBioscience 11B11 53-7041-82 10 µg/ml
anti-IL-13-PE-Cyanine7 Rat IgG1 Fisher Scientific eBio13A 15538636 1:200
anti-KLRG1-AF488 Hamster IgG2κ BD Pharmingen 2F1 562190 10 µg/ml
anti-CD3-AF532 eBioscience 17A2 58-0032-82 2 µg/ml
anti-CD45-FITC human IgG1 Miltenyi Biotec REA737 130-110-658 1:100

anti-CD45-VB human IgG1 Miltenyi Biotec REA737 130-110-802 1:200
anti-Siglec-F-PECy7 human IgG1 Miltenyi Biotec REA798 130-112-334 1:100
anti-CD11b-VG recombinant human IgG1 Miltenyi Biotec REA713 130-110-559 1:100
anti-B220-APC rat IgG2a Miltenyi Biotec RA3-6B2 130-102-259 1:100
anti-CD11c-VB, hamster IgG Miltenyi Biotec N418 130-102-797 1:100
anti-CD49b- BV421, Rat IgM BD Horizon Dx5 563063 1:50
anti-IgE-FITC, Rat IgG1 BD Pharmingen R35-72 553415 1:50
anti-CD125-PE, Rat IgG1 BD Pharmingen T21 558488 1:100
Purified NA/LE Rat Anti-Mouse CD124 BD Pharmingen mIL4R-M1 552288 10 µg/mL
anti-Siglec-F-Alexa647, Rat IgG2a BD Pharmingen E50-2440 562680 1:100
anti-CD131-PE Miltenyi Biotec REA193 130-118-456 1:100
anti-c-KIT APC eBioscience 2B8 17-1171-82 1:200
rabbit polyclonal HRP-conjugated anti-mouse IgG Invitrogen n/a 61-6520 1:5000
Goat polyclonal Anti-Mouse IgG1 Human ads-HRP Southern Biotech n/a 1070-05 1:4000
Goat anti-Rat IgG (H+L), Alexa Fluor 594 Invitrogen n/a A11007 1:150
Goat polyclonal Anti-Mouse IgG2a Human ads-HRP Southern Biotech n/a 1080-05 1:4000
goat polyclonal anti-mouse IgE antibody Bio-rad n/a STAR110 2 µg/mL
goat polyclonal HRP-conjugated anti-mouse IgG Bethyl Laboratories n/a A90-131P 1:5000
anti-IgE antibody coupled to AF488,
Rat IgG1, κ Biolegend RME-1 406910 10 µg/ml

Validation: Antibody validation was deferred to the manufacturers (including evaluation of purity, sensitivity, F/P ratio, specificity and lot-to-lot consistency).

Eukaryotic cell lines

Policy information about cell lines

Cell line source(s): CTLL-2 cells (ECACC, Ref. 93042610, batch number: 12K006)
HEK-Blue™ IL-4/IL-13 reporter gene cell line (InvivoGen, hkb-il413, batch number: X14-37-01)

Authentication: None of the cell lines were autheticated.

Mycoplasma contamination: Cell lines were not tested for mycoplasma contamination but no indication of contamination was observed.

Commonly misidentified lines (See ICLAC register): No commonly misidentified cell lines were used

Animals and other organisms

Policy information about studies involving animals; ARRIVE guidelines recommended for reporting animal research

Laboratory animals: Female BALB/cJRj mice at 5-6 weeks of age were purchased from Janvier Labs, and maintained in a specific pathogen-free facility at Institut Pasteur or Institut Jacques Monod. IL4RA/IL13/IL4 humanized mice were generated at University of North Carolina, USA and maintained in a specific pathogen-free facility at Institut Pasteur, France. Full description of the generation and characterization of IL4RA/IL13/IL4 mice are provided in the supporting information file, Figure 4 and Supplementary Figure 17.

Mice will be housed at Institut Pasteur in specific pathogen free conditions ensuring an appropriate lighting. Fluorescent light is provided with 14:10 h light:dark cycle (6:30 AM to 8:30 PM). Animal facility has central air conditioning equipment which maintains constant temperature of 22 ± 2°C. Air is renewed at least 20 times per hour in animal rooms. Mice will receive food and water ad libitum.

Wild animals: The study did not involve wild animals.

Field-collected samples: The study did not involve samples collected from the field.

Ethics oversight: All animal care and experimentation were conducted in compliance with the guidelines and specific approval of the Animal Ethics committee CETEA (Institut Pasteur, Paris, France) registered under #170043, and by the French Ministry of Research. The protocol also received the authorization number EU0285 - Institut Jacques Monod PHEA - APAFiS - Autor. APAFiS #165.

Note that full information on the approval of the study protocol must also be provided in the manuscript.

Flow Cytometry

Plots

Confirm that:

- [x] The axis labels state the marker and fluorochrome used (e.g. CD4-FITC).
- [x] The axis scales are clearly visible. Include numbers along axes only for bottom left plot of group (a 'group' is an analysis of identical markers).
- [x] All plots are contour plots with outliers or pseudocolor plots.
- [x] A numerical value for number of cells or percentage (with statistics) is provided.

Methodology

Sample preparation	Bronchoalveolar lavages (BALs) were performed 24 h after the last challenge with HDM in anesthetized mice (187.5 mg/kg ketamine and 18.75 mg/kg xylazine). After semi-excision of the trachea, a plastic canula was inserted, and airspace was washed with 1 ml of PBS containing 2.6 mM EDTA and 2.5 % (v/v) FBS. This operation was repeated for a total of 3 times. For the analysis of leukocytes in lung tissue, right lung lobes were harvested 24 h after the last challenge with HDM, and transferred into gentleMACS™ C tubes (Miltenyi) containing lung dissociation kit (Miltenyi). Tubes were attached upside down on a gentleMACS dissociator (Miltenyi). After a washing step, red blood cells were lysed with Ammonium Chloride Potassium (ACK) lysing buffer (Thermo Fisher scientific), and single cell suspensions were 0.22 µm-cut-off filtered. Blood was collected on heparin. Red blood cells were lysed with ACK lysis buffer (Thermo Fisher scientific). Cells were stained with anti-SiglecF-PECy7 (clone # REA798, Miltenyi), anti-CD49b-BV421 (clone Dx5, eBioscience), anti-IgE-FITC (clone # R35-72, BD Pharmingen) and anti-CD131-PE (clone #REA193, Miltenyi). Blood eosinophils were gated as SiglecF+, SSChigh, and blood basophils as CD49b+, CD131+, IgE+. To harvest peritoneal cells, 5 ml of PBS were injected into the peritoneal cavity and the abdomen was massaged gently for 20 seconds. Fluid containing peritoneal cells was collected.
Instrument	Data were acquired using Miltenyi MACSQUANT 10 and 16 or BD LSRFortessa™ cell analyzer (BD Biosciences).
Software	Data were analysed using FlowJo software (TreeStar).
Cell population abundance	All cell populations in bronchoalveolar lavage (BAL) fluid, lung single cell suspensions or blood leukocytes are represented as percentage of total leukocytes or numbers (with total leukocyte numbers provided)
Gating strategy	All gating strategies are detailed in the Supporting Information file. A figure exemplifying the gating strategy for lung-resident regulatory eosinophils is provided in the supporting information file.<br><br>Macrophages were gated as CD45+, CD11c+, Siglec-F+, CD11b+, B cells as CD45+, CD11c-, B220+, T cells as CD45+, CD11c-, CD3+, neutrophils as CD45+, CD11c-, B220-, CD3-, Ly6G+, CD11b+ and eosinophils as CD45+, CD11c-, B220-, CD3-, Ly6G-, Siglec-F+, SSChigh. Blood eosinophils were gated as Siglec-F+, SSChigh, and blood basophils as CD49b+, CD131+, IgE+.). Peritoneal mast cells were gated as c-KIT+, IgE+. Regulatory eosinophils were gated as CD45+, PI-, CD125+, Siglec-F int.

- [x] Tick this box to confirm that a figure exemplifying the gating strategy is provided in the Supplementary Information.
